# Electrical resistance of CNT-PEEK composites under compression at different temperatures

**DOI:** 10.1186/1556-276X-6-419

**Published:** 2011-06-13

**Authors:** Mohammad Mohiuddin, Suong Van Hoa

**Affiliations:** 1 Department of Mechanical and Industrial Engineering, Concordia Centre for Composites, Centre for Applied Research on Polymers and Composites (CREPEC), Concordia University, 1455 de Maisonneuve Blvd. W Montréal, Québec, Canada H3G 1M8

**Keywords:** Compression pressure, Carbon nanotubes, Polyether ether ketone (PEEK), electrical resistance, tunneling

## Abstract

Electrically conductive polymers reinforced with carbon nanotubes (CNTs) have generated a great deal of scientific and industrial interest in the last few years. Advanced thermoplastic composites made of three different weight percentages (8%, 9%, and 10%) of multiwalled CNTs and polyether ether ketone (PEEK) were prepared by shear mixing process. The temperature- and pressure-dependent electrical resistance of these CNT-PEEK composites have been studied and presented in this paper. It has been found that electrical resistance decreases significantly with the application of heat and pressure.

## Introduction

Electrical conductivity of thermoplastic composites containing carbon nanotubes (CNTs) is due to the formation of a continuous conductive network in the polymer matrix [1]. This network consists of specific spatial arrangement of conductive elements so that low resistance electrical paths are developed for free movement of electrons. Enhancement of electrical conductivity of polymer by mixing them with multiwalled carbon nanotubes has found significant applications in newer areas such as electrostatic charge dissipation, electronic equipment, pressure sensors, sensor of vehicle weight in highways, selective gas sensors, and strategic materials such as EMI/RFI shielding in computer and cellular phone housing etc. [2-4].

The electrical resistance of conductive polymeric composites changes with externally applied heat and pressure [5,6]. Surveying of literature shows that most researchers so far explored the applicability of pressure sensors made of carbon black, carbon fiber, CNT, metallic powders, graphite, etc. as conducting element and elastomeric rubber materials like NBR, SBR, EPDM etc. as matrix [7-10]. Limited work has been done on the possibility of using advanced thermoplastic materials, e.g., PEEK, PMMA as matrix in manufacturing pressure sensing element.

## Experimental

### Materials

Polyether ether ketone (PEEK) powder of grain size 80 μm purchased from Good Fellow, England was used as polymer matrix and multiwalled carbon nanotubes (CNT) Baytubes C 150 P (C-purity ≥95 wt.%, length > 1 μm, diameter 4-13 nm, synthesized by chemical vapor deposition) purchased from Bayer MaterialScience, Leverkusen, Germany were used as the filler in this study. Both PEEK and carbon nanotubes were used "as received" to fabricate the samples.

### Sample preparation and testing

The melting and high temperature shear mixing was done in a laboratory scale Torque Rheometry system Brabender Intelli-Torque Plasti-Corder (type IT 7150) at mixing temperature of 380°C, rotor speed of 100 rpm, and mixing time 20 min. High shear mixing is usually carried out when the nanoparticles are in solid and the polymer matrix is in liquid or powder form [11]. Under these conditions, high shear mixing breaks the nanoparticle aggregates and disperses the nanoparticles into the polymer matrix. To achieve uniform dispersion of nanotubes, helical-shaped twin screw extruders were used in the mixing machine. Different weight percentages of CNT were mixed with PEEK. The CNT/PEEK melt was then molded in a Wabash compression molding machine at melting temperature of PEEK (340°C) with compaction pressure of 10 tons and holding time 15 min using a mold made of 1.4-mm thick stainless steel plate with six holes of 25.4 mm diameter. This produces six round-shaped samples having 25.4 mm diameter and 1.4 mm thickness at one time. After cooling and solidification, the samples were polished by 400 series sand paper and tested for electrical properties.

### Electrical resistance measurement

The electrical resistance measured by Fluke digital multimeter *R*_measured _consists of following three components:

The electrical volume resistivity of the composites was measured using a high resistance meter (Model 4339B, Agilent, Santa Clara, CA, USA). From volume resistivity and geometry of the sample, the actual sample electrical resistances (*R*_sample_) were calculated using the equation  where *t *is the thickness, *A *is the cross sectional area, *ρ *is the volume resistivity of the sample.

**Figure 1:**
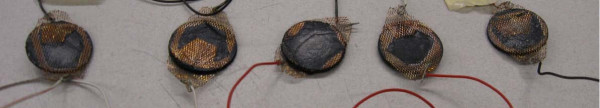
**Pictures of CNT-PEEK samples**.

Metallic hook was connected to a highly conductive copper wire of short length (about 300 mm) so that magnitudes of the component *R*_wires _is much smaller than the other terms and can be ignored. Contact resistance (*R*_contact_) plays a significant role relative to the overall specimen resistance. Contact resistance depends on contact area, contact gap, type of junction (metallic/metallic or metallic/semiconducting) etc. Conductive silver paint [12] is commonly used to minimize the contact resistance at electrodes. In our case, under application of pressure and temperature, the contact points are expanded under compression plate which may affect the measurement of actual sample resistance. To overcome this situation and to get repeatable result, we impregnated the conductive copper mesh on both surfaces of the samples (Figure 1) by pressing them in the Wabash hot press at 340°C for 1 min with a small compaction pressure of 0.5 ton. To impregnate the copper mesh onto the round shaped CNT-PEEK sample, a very thin film of same percentage of CNT and PEEK was used on top and bottom of the sample so that the copper mesh is impregnated permanently and does not move laterally during the compression experiment. With this arrangement, the contact resistance does not change under application of compression and temperature. As such, for comparison purposes, the effect of the contact resistance on different samples can be factored out. Electrical wires are connected to the copper meshes for electrical resistance measurement.

To measure the electrical resistance at elevated temperatures under compression, the entire electrode system was placed in a confined aluminum heater where the temperature could be monitored and controlled over the range 20-500°C. Heat was supplied to the sample by a programmable i-series temperature/process controller purchased from Omega Engineering Inc., Stamford, CT, USA. Electrical resistance was measured while compression pressure was applied using MTS testing machine. The samples were compressed by applying a pressure along the thickness direction from 0 to 40 MPa with increments of 2 MPa. Temperature was increased simultaneously from 40°C to 140°C with increments of 10°C. Each pressure and temperature level was kept constant for 5 min to get stable readings of sample resistance. At a constant temperature and pressure, the sample resistance was measured across the thickness of the sample by using a Fluke digital multimeter, which can measure resistances up to 100 MΩ.

## Results and discussion

The experiments were performed for at least three samples for each of the 8%, 9%, and 10% CNTs. *R*_measured _and *R*_sample _(obtained by calculation from resistivity data) at zero pressure and room temperature are presented in Table 1. The difference between measured and calculated resistances is less than 8%. This can be due to contact resistance and to variability in sample to sample and experimental errors. This degree of error can be used to indicate the degree of accuracy of the results.

**Table 1:** Comparison of electrical resistance *R*_sample _and *R*_measured _at 0 pressure and *T*_room_

Weight percent of CNTs (%)	*R*_sample_	*R*_measured_	Percentage of difference (%)
8	3,120 Ω	3,341 Ω	6.6
9	2,505 Ω	2,670 Ω	6.2
10	2,109 Ω	2,280 Ω	7.5

### Effect of temperature

**Figure 2:**
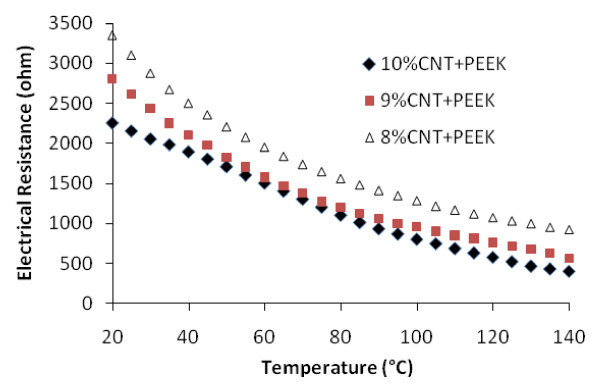
**Comparison of electrical resistance at different temperatures and at zero pressure**.

Figure 2 shows the effect of temperature on electrical resistance at no applied pressure. The following can be observed:

• Higher amount of CNT gives lower electrical resistance. The effect of the amount of CNT is more at lower temperature than at higher temperature.

• Increasing temperature reduces the electrical resistance (negative temperature coefficient, or NTC)

• At 10% CNT, the curve is close to that of a straight line. The curves are nonlinear for 9% CNT and 8% CNT. The effect of increasing temperature on reduction in electrical resistance is more at lower temperature range (from 20°C to 70°C) than at higher temperature range (from 70°C to 140°C).

The terminology used to indicate the reduction in electrical resistance due to temperature increase is NTC. The opposite effect is positive temperature coefficient (PTC). The symbol OTC can be used to indicate no temperature effect on resistance. Whether any of these effects occurs depends on the nature of the polymer, the filler, and the concentration of the filler. PTC effect has been reported by many researchers for carbon fiber-filled elastomeric composites [7], Carbon black-polyethylene (PE) composites [13,14], short carbon fiber-filled LMWPE-UHMWPE composites [15], multiwalled CNT-filled high-density PE composites [16]. On the other hand, NTC effect has also been reported for carbon black-low density PE composites [17], multiwalled CNT-polyurethane (PU) composites [18], acetylene carbon black-filled systems [19] etc. The PEEK/CNT in this study shows NTC effect. This effect is stronger at the lower temperature range than at high temperature range.

### Effect of temperature and pressure

**Figure 3:**
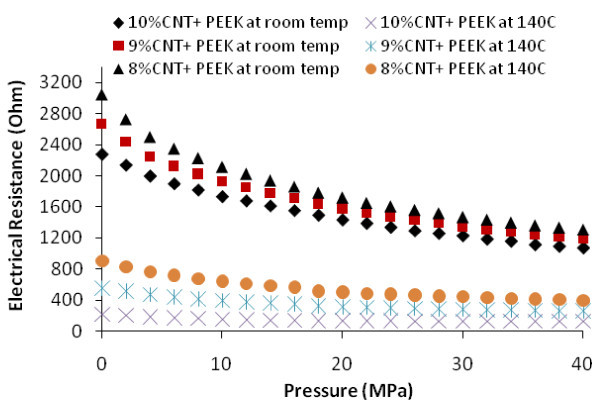
**Electrical resistance vs. pressure at room temperature and 140°C**.

Figure 3 shows the effect of both temperature and pressure. Note that the same three samples were used to produce the results in Figures 2 and 3. The results in Figure 2 were obtained first. For example, the sample with 8% CNT was heated to 140°C while the resistances were measured. This sample was then cooled down, and pressure and temperature were applied to produce the results shown in Figure 3. The resistance values at room temperature and zero pressure in Figure 3 are slightly larger than those in Figure 2. For example, for 8% CNT, this value in Figure 3 is about 3,000 Ω while that in Figure 2 is about 3,300 Ω. This can be due to irreversible changes in the conducting networks caused by the initial heating process [7] which induces some residual conductivity. In Figure 3, two sets of curves are shown. The upper set of curves presents the results for room temperature, while the lower ones for 140°C. The following can be observed:

• Increasing the pressure reduces the electrical resistance. The effect of pressure is more at room temperature than at 140°C.

• At room temperature, the effect of pressure is more in the lower pressure range (from 0 to 20 MPa) and there is almost no pressure effect at higher pressure (more than 20 MPa)

• There is almost no effect of pressure on the electrical resistance at 140°C, particularly for higher CNT loadings (9% and 10%).

### Explanation for the effect of temperature and pressure on electrical resistance of CNT/polymer composites

The effect of temperature and pressure on the electrical resistance of CNT/polymer composites may be explained based upon two main mechanisms responsible for electrical conductivity (or electrical resistance) in CNT/polymer composites.

• Particle contacts - conduction by electron transport. The contacts between the different carbon nantubes provide the circuit for electrons to flow. At the percolation threshold, there is just sufficient contact for the material to be conductive. Above the percolation threshold, parameters that affect the number of contacts are:

◦ Amount of fillers. More CNTs, more contacts, and lower electrical resistance. This is evident in Figures 2 and 3.

◦ More compression. Compression squeezes the CNTs together, giving better probability for contacts (Figure 3).

◦ There is a saturation phenomenon for both the amount of fillers and the level of compression. This means that the rate of reduction of electrical resistance is more at lower levels of CNT and compression and the rate reduces as the levels of fillers or compression are increased. This is because once full electrical conductivity is established; it is difficult to increase it.

◦ Aspect ratio of fillers. The aspect ratio of the fillers has important influence on the electrical resistance. Larger aspect ratio reduces electrical resistance. Ansari et al [20] studied the electrical conductivity of PVDF reinforced with two types of fillers. They found that Functionalized Graphene sheet (FGS)-PVDF system exhibited NTC while exfoliated graphite (EG)-polyvinylidene fluoride (PVDF) system exhibits PTC. The explanation given is that FGS has higher aspect ratio than EG.

• Conduction by electron tunneling. In addition to conduction by electron transport across contact points, conductivity in CNT/polymer system also occurs by electron tunneling across gaps between the CNTs. Conduction by electron tunneling depends on the length of the gap between the CNTs. The longer is the gap, the more difficult is the electron tunneling, and the larger is the electrical resistance. Parameters that affect the electron tunneling are:

◦ The relative dominance between the number of contacts and the gaps between the CNTs. If the number of contacts is dominant then increase in temperature would increase in electron activity and this would reduce the electrical resistance. There should be a critical amount of contacts beyond which the gaps between the CNTs would become irrelevant.

◦ The stiffness of the polymer material. In situations where there is a relatively small amount of fillers, the stiffness of the polymer material plays an important role. For material with higher stiffness, increasing in temperature may not produce in large deformation of the gaps between CNTs, while the opposite holds true for material with lower stiffness. Work done in references [7,13-16] showed PTC. These experiments were performed above the glass transition temperature (*T*g) of the polymers (*T*_g _of Elastomer -70°C, PE -120°C, PVDF -35°C). Our investigation for CNT-PEEK composites was carried out below glass transition temperature, *T*_g _(*T*_g _of PEEK is 146°C) and we obtained NTC. However Figure 2 shows that the NTC effect decreases with increasing temperature, due to the softening of the polymer at higher temperature.

The change in electrical resistance with applied pressure can be explained by considering several phenomena that happens simultaneously in the composite system: breakdown of existing conductive paths, formation of new conductive paths and change or redistribution of conductive paths [21]. Formation of this conducting path occurs by direct contact between electrically conductive CNTs and when the inter particle distance between CNTs is only few nanometers. There exists a threshold value of 1.8 nm [22] for this inter particle gap at which electrons can easily jump across the gap (electron tunneling). Application of high pressure reduces this electron tunneling gap, thereby leading the composites to exhibit high conductivity at high applied pressure.

## Conclusion

Electrically conductive CNT reinforced PEEK composites were manufactured and effect of temperature and pressure on the electrical resistance was studied. Negative temperature coefficient of resistivity (NTC effect) has been noticed in the case of CNT-PEEK composites over a temperature range from room temperature to 140°C. Application of pressure also reduces the electrical resistance. The explanation for this behavior was given based on two main mechanisms responsible for the electrical conductivity of CNT/polymer composites. This relates to the influence of the amount of fillers, the aspect ratio of the fillers and the stiffness of the matrix.

## Competing interests

The authors declare that they have no competing interests.

## Authors' contributions

MM: (i) has made substantial contributions to conception and design (ii) prepared the sample and did the experiment (iii) did analysis and interpretation of experimental data (iv) drafted the manuscript.

SVH: (i) has made substantial contributions to conception and design (ii) revised the manuscript critically for important intellectual content (iii) has given final approval of the version to be published.
